# Respiratory patterns and baroreflex function in heart failure

**DOI:** 10.1038/s41598-023-29271-y

**Published:** 2023-02-08

**Authors:** Alberto Radaelli, Giuseppe Mancia, Giulia Balestri, Daniela Bonfanti, Paolo Castiglioni

**Affiliations:** 1 Division of Cardiac Rehabilitation, Fondazione IRCCS San Gerardo dei Tintori, Via Pergolesi, 33, 20900 Monza, MB Italy; 2 University of Milano-Bicocca, Milan, Italy; 3 Laboratory of Movement Analysis and Bioengineering of Rehabilitation (Lamobir), IRCCS Fondazione Don Carlo Gnocchi ONLUS, 20148, Milan, Italy; 4 Department of Biotechnology and Life Sciences (DBSV), Università degli Studi dell′Insubria, 21100 Varese, Italy

**Keywords:** Cardiology, Cardiovascular diseases

## Abstract

Little is known on the effects of respiratory patterns on baroreflex function in heart failure (HF). Patients with HF (n = 30, age 61.6 ± 10 years, mean ± SD) and healthy controls (CNT, n = 10, age 58.9 ± 5.6 years) having their R–R interval (RRI, EKG), systolic arterial blood pressure (SBP, Finapres) and respiratory signal (RSP, Respitrace) monitored, were subjected to three recording sessions: free-breathing, fast- (≥ 12 bpm) and slow- (6 bpm) paced breathing. Baroreflex sensitivity (BRS) and power spectra of RRI, SBP, and RSP signals were calculated. During free-breathing, compared to CNT, HF patients showed a significantly greater modulation of respiratory volumes in the very-low-frequency (< 0.04 Hz) range and their BRS was not significantly different from that of CNT. During fast-paced breathing, when very-low-frequency modulations of respiration were reduced, BRS of HF patients was significantly lower than that of CNT and lower than during free breathing. During slow-paced breathing, BRS became again significantly higher than during fast breathing. In conclusion: (1) in free-breathing HF patients is present a greater modulation of respiratory volumes in the very-low-frequency range; (2) in HF patients modulation of respiration in the very-low and low frequency (around 0.1 Hz) ranges contributes to preserve baroreflex-mediated control of heart rate.

## Introduction

Evidence is available that slowing of respiration increases the sensitivity of the baroreflex^1,2^, with, as a result, an increase of vagal influences on the heart and a reduction of sympathetic activity^3,4^. This should play a favorable role in heart failure (HF) in which the frequently reported presence of slow deep breaths should improve baroreflex sensitivity from the marked impairment typical of this condition^5,6^. The improved baroreflex function in turn should lead to an increase in vagal tone as well as a reduction of sympathetic tone, lessening the autonomic abnormalities that reduce the survival of HF patients^7^. However, whether and to what extent slow breathing exerts these effects in HF is not completely clear because HF activates a number of factors that can mask the influence of slow breathing on the baroreflex, and variably so according to the HF severity. For example, in HF reduced perfusion and hypoxia of the carotid and aortic bodies^8,9^ can stimulate chemoreceptor reflexes which have been found to oppose and even completely neutralize the baroreflex influences on the cardiovascular system^10,11^.

The present study was performed to analyze the effects that different ventilation patterns have on the modulation exerted by the baroreflex on the heart and the peripheral circulation. The autonomic nervous system was studied by noninvasive spectral analysis of cardiovascular signals (heart rate by EKG-derived RR interval and systolic blood pressure by beat-to-beat measurement) and respiration. The effects of different patterns of respiration on baroreflex sensitivity and cardiovascular variability were assessed by studying spontaneous breathing, fast-paced breathing and slow-paced breathing, in each condition having matched healthy controls for comparisons.

## Methods

The study followed the guidelines of the Declaration of Helsinki, its protocol was approved by the Ethics Committee of the S. Gerardo Hospital (Monza) and the University of Milano Bicocca with ID code RESPSCOMP. Informed consent was provided from all the participants.

### Subjects

We enrolled 30 consecutive patients with HF due to ischemic cardiomyopathy and 10 age-matched healthy volunteers as controls (CNT). Each participant was subjected to a clinical assessment that included physical examination, urinalysis, blood chemistry, baseline and exercise EKG, and an echocardiogram. In HF patients venous blood was withdrawn to measure plasma catecholamine concentration (noradrenaline and adrenaline) and level of N-terminal prohormone of brain natriuretic peptide (NT-proBNP). The normal plasma noradrenaline and adrenaline values were considered to be, respectively, between 95–446 and 10–67 pg/ml while the normal value of NT-proBNP was considered to be < 300 pg/ml^12–14^. Plasma catecholamines and NT-proBNP were not measured in healthy controls for ethical reasons.

### Protocol

Participants were asked to refrain from smoking and drinking coffee or tea for 24 h before the study. The experimental session took place in the early afternoon, with the subjects supine in a quiet room at an environmental temperature between 20 and 23 °C. The recordings started after 20–30 min of rest. Participants were allowed to breathe spontaneously during the first 15 min of the recording and then were asked to breathe following a metronome. Two paced frequencies, fast and slow, were set and allocated in random order. Aim of the fast-paced breathing was to remove the spontaneous breath-by-breath variability during the breathing cycle while preserving the physiological average breathing rate: thus, the paced respiratory frequency was set at a constant rate between 15 and 12 bpm (according to the patient's preference), for 5 min. Aim of the slow-paced breathing was to evaluate the influence of a 10-s breathing cycle on cardiovascular variability: thus, the respiratory rate was paced at 6 bpm for 4 min. In both patients and controls the recorded signals included the continuous noninvasive finger blood pressure (Finapres, Ohmeda Inc., Englewood, Colorado, USA) and one-lead EKG, sampled at 200 Hz. The recorded signals also included the uncalibrated respiratory volume (RSP) by induction plethysmography (Respitrace, Ambulatory Monitoring, Ardsley, NY)^15^ but due to a technical problem, this device was available for the experimental sessions of all the 10 controls and 17 HF patients. Oxygen saturation (SaO_2_) was measured at the end of each (free, fast-paced, and slow-paced) breathing condition in all participants.

### Data analysis

**Figure 1:**
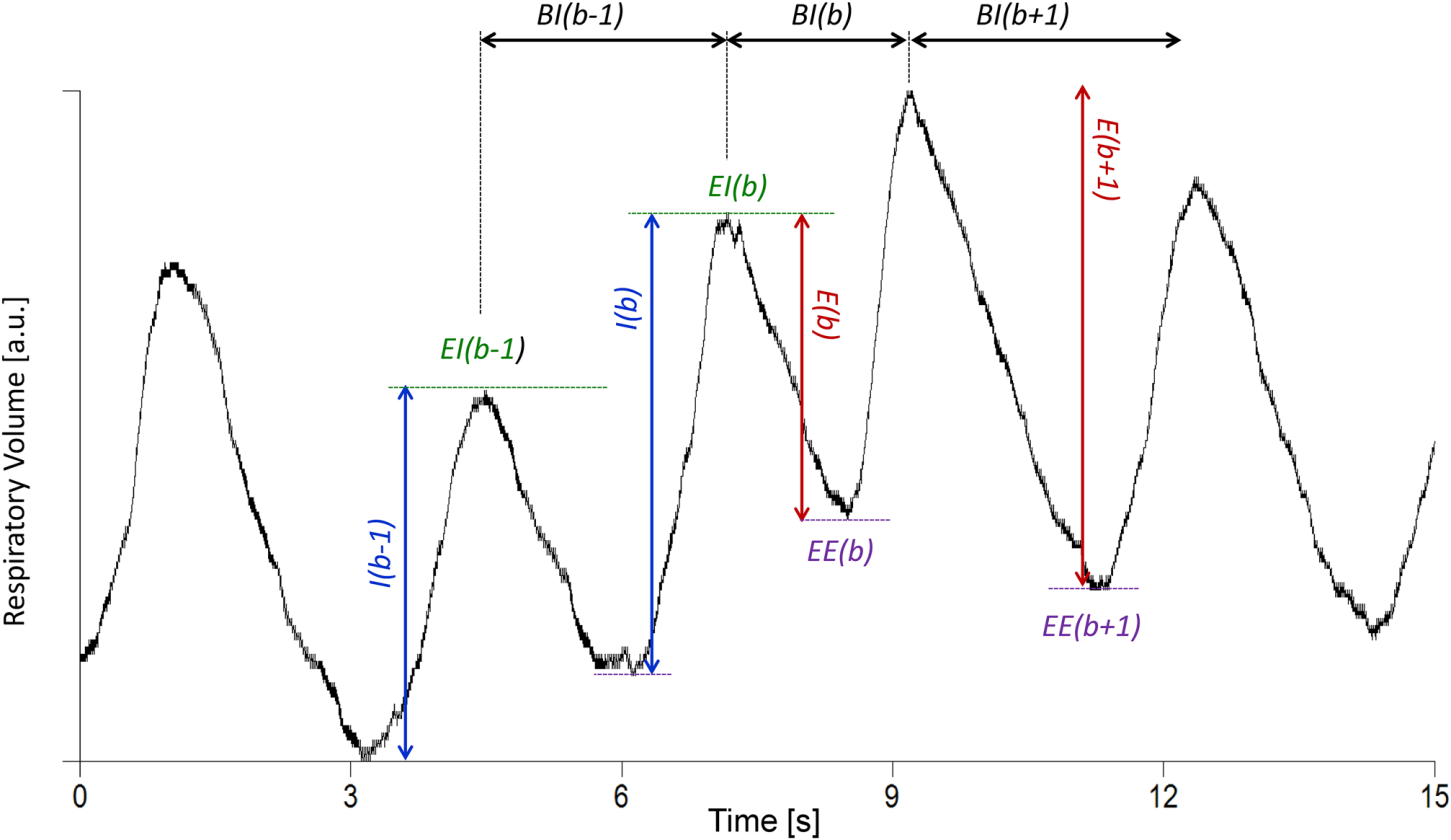
Breath-by-breath respiratory series. From the respiratory volume sampled at 200 Hz (continuous black line) we extracted 5 breath-by-breath series: the end-inspiratory (EI, green dashed line) and end-expiratory (EE, purple dashed line) volumes; the inspiratory (I, blue arrow) and expiratory (E, red arrow) volumes; and the breathing interval (BI, black arrow). For a given breath *b*, the EI volume is the maximum respiratory volume within the breath; the EE volume is the minimum respiratory volume between the EI volumes of breaths *b* and *b* + 1; I volume is the difference between the EI volume of the breath *b* and the EE volume of the breath *b* − 1 while the E volume is the difference between the EI and EE volumes of the same breath *b*; finally, BI is the time interval between consecutive EI volumes.

The R–R interval (RRI) series were calculated beat-by-beat as the distance between consecutive peaks of the R wave of the EKG, identified by a derivative-and-threshold algorithm with parabolic interpolation to refine the R-wave fiducial point^16^. The average standard deviation was calculated over running windows of 3 min (SDNN) as an index of overall variability. The percentage of intervals at least 50 ms longer or shorter than their preceding interval, pNN50, was calculated as an index of vagal heart rate control^16^. The beat by beat series of systolic blood pressure (SBP) was identified from the finger arterial pressure. Artifacts and ectopic beats were removed. A zero-crossing algorithm identified the start and end of each breath in the respiratory signal. The series of the end-expiratory (EE) and end-inspiratory (EI) volumes, inspiratory (IV) and expiratory volumes (EV), and breathing intervals (BI), were derived breath-by-breath from the respiratory signal as in Fig. 1.

Before spectral analysis, the beat-by-beat and breath-by-breath series were interpolated and resampled at 5 Hz while RSP was decimated at 5 Hz. The power spectral densities of RSP, RRI, and SBP series, as well as of the respiratory volumes and intervals, were calculated by the Welch periodogram with 50% overlapped Hann running windows of 180 s length and broadband spectral smoothing^17^. The power spectra of the uncalibrated respiratory volumes were normalized to have total power equal to one. Power spectral densities were integrated between 0.005 and 0.04 Hz to obtain the very-low-frequency power (VLF_p_), between 0.04 and 0.15 Hz to obtain the low-frequency power (LF_p_), and between 0.15 and 0.40 Hz to obtain the high-frequency power (HF_p_). During fast-paced breathing, HF_p_ of RRI is an index of vagally driven respiratory oscillations of heart rate while during slow-paced breathing, LF_p_ of RRI is an index of respiratory heart-rate oscillations driven by both cardiac sympathetic and vagal outflows^16^.

Coherency spectra between RRI and SBP, between RSP and RRI, and between RSP and SBP, were estimated from the corresponding spectra and cross-spectra^17^. Two indexes were derived from the squared coherence modulus: the 10-s coherency, as the average over the 0.08–0.12 Hz range, and the 25-s coherency, as the average over the 0.03–0.05 Hz range. These frequency bands correspond to the peak with a period of around 10 s and to the valley with a period of around 25 s that characterize the SBP–RRI coherency. The SBP–RRI coherency peak at 10 s (k_SBP–RRI_ at 10 s) was taken as an index of the baroreflex function, reflecting the baroreflex coupling between blood pressure and heart rate at the frequency of Mayer waves. Because the effect of the SBP–RRI cross-coupling around 25 s is minimal, the 25 s coherency between RSP and SBP (k_RSP–SBP_ at 25 s) was taken as an index of respiratory-blood pressure coupling whereas the 25 s coherency between RSP and RRI (k_RSP–RRI_ at 25 s) was taken as an index of the respiratory–heart rate coupling.

The spontaneous baroreflex sensitivity, BRS, was estimated with the transfer function approach^18^ by calculating the average of the SBP–RRI transfer function over the LF band and considering only the spectral lines with a coherency modulus greater than 0.5 and a negative phase between SBP and RRI.

### Statistics

General characteristics were compared between groups by the Mann–Whitney test (ordinal data) or the Fisher's exact test (categorical data). Broadband power spectra were compared between groups by the Student's t statistic after log-transformation to obtain Normal distributions^19^. Coherency spectra were compared between groups by the Student's t statistic after Fisher z-transform to obtain Normal distributions^20^. Surrogate time series were generated from the RRI, SBP, and RSP series as described previously^21^. The surrogate series remove the coupling among RRI, SBP, and RSP while preserving the power spectrum and probability density function of the original series. Coherency spectra of surrogate and original series were compared by a one-sided paired t-test to identify the frequencies where the squared coherence modulus of the original series was significantly greater than zero.

Cardiovascular and respiratory indexes were compared between groups and conditions by repeated-measures ANOVA. When the factor “group”, or “time”, or their interaction revealed statistical trends (p < 0.10), differences between conditions and groups were tested by applying the Least-Significant Difference correction for multiple comparisons. Power spectra were log-transformed and coherency indexes were z-transformed. When the distributions did not pass the Shapiro–Wilk normality test at p = 0.05, differences were assessed by ANOVA on ranks. The level of statistical significance was set at p < 0.05. Statistics were performed with the STATISTICA 6.0 Software (StatSoft, Inc., Tulsa, OK, USA).

## Results

### Patients characteristics

As shown in Table 1, HF patients and healthy controls were not significantly different in gender, age, and body mass index. This was the case also for heart rate and blood pressure values. As expected HF patients had a significantly lower ejection fraction compared to controls. NT-proBNP levels exceeded the normal range in 80% of HF patients while this happened in 26.7% and 23.3% of the patients, respectively for plasma noradrenaline and adrenaline (Table 1). During free breathing, the respiratory rate of HF patients was not significantly different from that of controls (Table 2). There were no differences between groups in SaO_2_, the mean (SD) value being 97.5% (1.3%) in CNT and 97.0% (0.9%) in HF (p = 0.25) during free breathing; 97.8% (1.1%) in CNT and 98.1% (1.1%) in HF (p = 0.47) at the end of fast-paced breathing; and 98.4% (1.4%) in CNT, 98.4% (0.9%) (p = 0.65) in HF at the end of slow-paced breathing.

**Table 1:** General data on patients and controls with significance p of their difference.

	CNT	HF	p value
Males/females	10/0	27/3	0.56
Age (year)	58.9 (5.6)	61.6 (10.2)	0.30
Body Mass Index (kg/m^2^)	24.5 (2.1)	25.8 (3.3)	0.41
Systolic BP (mmHg)	121.4 (13.5)	112.6 (13.8)	0.14
Diastolic BP (mmHg)	75.7 (5.3)	70.5 (6.7)	0.09
Heart rate (bpm)	66.7 (8.8)	65.1 (12.2)	0.29
SDNN (ms)	32.9 (10.1)	27.8 (13.8)	0.11
Ejection fraction (%)	59.1 (2.6)	37.5 (7.0)	< 0.00001
NT-proBNP	na	885 (1168)	
Adrenaline (pg/mL)	na	43.2 (26.5)	
Noradrenaline (pg/mL)	na	411 (170)	
Medical therapy			
Bisoprolol	0	20	
Carvedilol	0	10	
Ace inhibitors	0	16	
AT1 antagonists	0	14	
Diuretics	0	20	

Values as mean (SD); p after Fisher’s exact test for sex composition, otherwise after Mann Whitney U test; *BP* blood pressure in sitting position by an arm cuff, *heart rate and SDNN* in supine position, *NT-proBNP* N-terminal prohormone of brain natriuretic peptide, *CNT* controls, *CHF* congestive heart failure patients.

### Vagal indexes of heart rate variability

**Figure 2:**
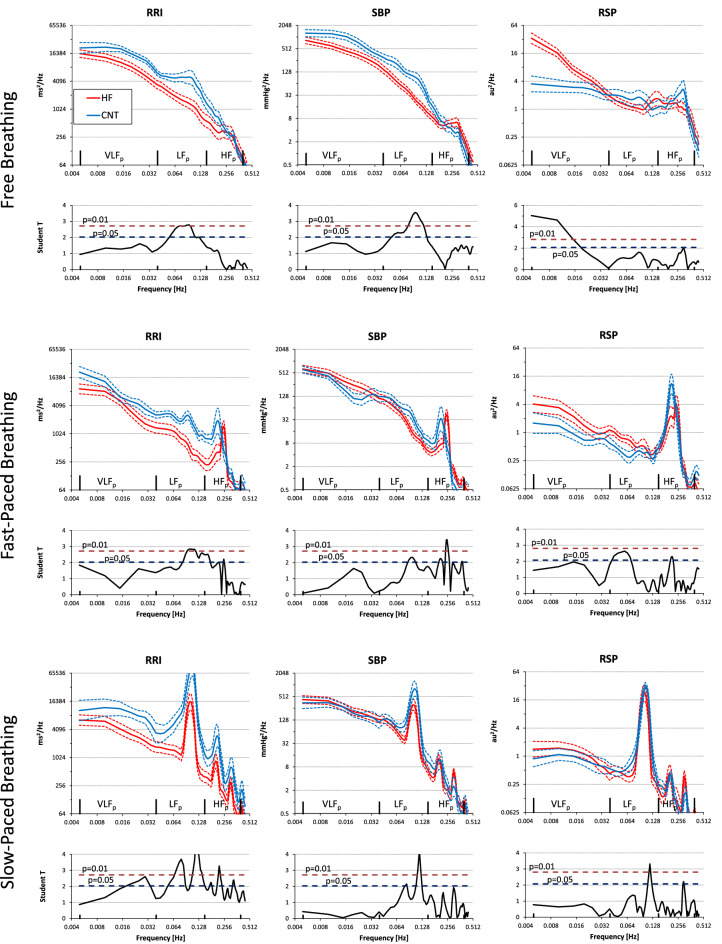
Power spectra during free, fast-paced, and slow-paced breathing. Power spectra in heart failure (HF) patients and controls (CNT) are shown as geometric mean ± geometric sem separately for R–R intervals (RRI), systolic blood pressure (SBP), and respiration (RSP). Panels below each couple of CNT and HF spectra show the Student T statistics between groups at each frequency, with horizontal dashed lines indicating the 0.05 and 0.01 significance thresholds: this means that T-values above the dashed lines point out differences between HF and CNT groups significant at the corresponding significance threshold. Frequency bands defining the very-low frequency power (VLF_p_), low-frequency power (LF_p_) and high-frequency power (HF_p_) are shown. Note the lower SBP and RRI powers around 0.1 Hz and the greater VLF_p_ of RSP for the HF group during free breathing.

During free and fast-paced breathing pNN50 was not significantly different in HF patients and healthy controls (Table 2). However, in controls pNN50 was significantly higher during slow-paced breathing than during fast-paced breathing while this was not the case for HF patients; thus, during slow-paced breathing pNN50 was significantly higher in controls than in HF patients (Table 3). Similarly, the power of respiratory-related RRI oscillations was not significantly different in the two groups during fast-paced breathing (HF_p_, Fig. 2; Table 2) but higher in controls than in HF patients during slow-paced breathing (LF_p_, Fig. 2; Table 3).

**Table 2:** Cardiovascular and respiratory indexes in free vs. fast-paced breathing by group, with factors significance.

	Group→	CNT	HF	Factors p value		
	Time↓			Time	Group	Time × group
RRI						
Mean (ms)	Free	917 (125)	951 (153)	0.012	0.47	0.85
	Fast	882 (136)	921 (134)*			
VLF_p_ (ms^2^)	Free	406 (1.1)	264 (1.2)	< 10^–4^	0.15	0.69
	Fast	219 (1.2)**	127 (1.2)**			
LF_p_ (ms^2^)	Free	442 (1.3)	170 (1.2)^#^	< 10^–5^	0.023	0.99
	Fast	218 (1.1)**	84 (1.3)**^,#^			
HF_p_ (ms^2^)	Free	110 (1.2)	90 (1.3)	< 10^–2^	0.46	0.32
	Fast	211 (1.3)*	125 (1.3)			
pNN50	Free	0.015 (0.031)	0.020 (0.136)	0.91	0.77	0.88
	Fast	0.031 (0.060)	0.039 (0.140)			
SBP						
Mean (mmHg)	Free	115.4 (16.1)	113.1 (21.7)	0.68	0.46	0.24
	Fast	120.5 (24.9)	110.7 (26.9)			
VLF_p_ (mmHg^2^)	Free	18.9 (1.2)	11.7 (1.2)	< 10^–3^	0.83	0.027
	Fast	6.7 (1.3)**	9.2 (1.3)			
LF_p_ (mmHg^2^)	Free	10.3 (1.3)	4.4 (1.2)^#^	0.022	0.07	0.10
	Fast	5.8 (1.2)*	4.0 (1.2)			
HF_p_ (mmHg^2^)	Free	1.3 (1.2)	1.6 (1.2)	< 10^–4^	0.73	0.42
	Fast	3.3 (1.4)**	3.2 (1.2)**			
Baroreflex function						
BRS (ms/mmHg)	Free	6.32 (3.25)	5.06 (7.43)	0.021	0.15	0.65
	Fast	5.76 (1.50)	4.29 (4.58)**			
k_SBP–RRI_ at 10 s	Free	0.695 (0.135)	0.569 (0.270)^#^	0.17	< 10^–2^	0.10
	Fast	0.797 (0.140)	0.560 (0.303)^##^			
RSP						
Breathing interval (s)	Free	4.7 (2.0)	4.4 (1.3)	0.78	0.55	0.79
	Fast	4.5 (0.3)	4.4 (0.4)			
VLF_p_ (au^2^)	Free	0.09 (1.28)	0.22 (1.16)^#^	< 10^–5^	0.029	0.43
	Fast	0.03 (1.22)**	0.06 (1.30)**			
LF_p_ (au^2^)	Free	0.18 (1.37)	0.16 (1.22)	< 10^–4^	0.39	0.25
	Fast	0.04 (1.18)**	0.05 (1.19)**			
HF_p_ (au^2^)	Free	0.53 (1.20)	0.45 (1.10)	< 10^–3^	0.13	0.71
	Fast	0.90 (1.02)**	0.77 (1.08)**			
Respiratory–cardiovascular coupling						
k_RSP–RRI_ at 25 s	Free	0.436 (0.194)	0.318 (0.278)	0.27	0.011	0.50
	Fast	0.499 (0.208)	0.336 (0.111)^#^			
k_RSP–SBP_ at 25 s	Free	0.453 (0.156)	0.260 (0.158)^#^	0.06	0.036	0.23
	Fast	0.440 (0.187)	0.391 (0.187)*			

Significance by ANOVA and by ANOVA on ranks for BRS and pNN50; *post-hoc* analysis by Fisher's LSD: the * and ** indicate significant differences between times at p < 0.05 and < 0.01; the ^#^ and ^##^ indicate significant differences between groups at p < 0.05 and < 0.01; RRI mean, SBP mean and breathing interval as mean (SD); powers as geometric mean (geometric SD); pNN50 and BRS as median (IQR).

**Table 3:** Cardiovascular and respiratory indexes in slow- vs. fast-paced breathing by group, with factors significance.

	Group→	CNT	HF	Factors p-value		
	Time↓			Time	Group	Time × Group
RRI						
Mean (ms)	Slow	919 (101)	941 (134)	< 10^–3^	0.52	0.29
	Fast	882 (136)**	921 (134)**			
VLF_p_ (ms^2^)	Slow	388 (1.2)	137 (1.2)^#^	< 10^–2^	0.044	0.022
	Fast	219 (1.2)**	127 (1.2)			
LF_p_ (ms^2^)	Slow	3143 (1.3)	499 (1.4)^##^	< 10^–11^	< 10^–2^	0.049
	Fast	218 (1.1)**	84 (1.3)**			
HF_p_ (ms^2^)	Slow	253 (1.4)	75 (1.3)^#^	0.32	0.09	0.038
	Fast	211 (1.3)	125 (1.3)**			
pNN50	Slow	0.194 (0.206)	0.062 (0.157)^#^	< 10^–4^	0.30	< 10^–2^
	Fast	0.031 (0.060)**	0.039 (0.140)			
SBP						
Mean (mmHg)	Slow	120.0 (30.8)	118 (23.8)	0.29	0.52	0.23
	Fast	120.5 (24.9)	110.7 (26.9)			
VLF_p_ (mmHg^2^)	Slow	7.7 (1.2)	8.5 (1.2)	0.86	0.53	0.56
	Fast	6.7 (1.3)	9.2 (1.3)			
LF_p_ (mmHg^2^)	Slow	29.9 (1.3)	13.6 (1.2)^#^	< 10^–11^	0.078	0.24
	Fast	5.8 (1.2)**	4.0 (1.2)**			
HF_p_ (mmHg^2^)	Slow	1.3 (1.4)	1.1 (1.2)	< 10^–5^	0.78	0.74
	Fast	3.3 (1.4)**	3.2 (1.2)**			
Baroreflex function						
BRS (ms/mmHg)	Slow	8.76 (1.77)	5.34 (6.04)^##^	< 10^–2^	0.011	0.31
	Fast	5.76 (1.5)*	4.29 (4.58)*			
k_SBP–RRI_ at 10 s	Slow	0.968 (0.042)	0.892 (0.13)^##^	< 10^–11^	< 10^–3^	0.50
	Fast	0.797 (0.140)**	0.560 (0.303)**^,##^			
RSP						
Breathing interval (s)	Slow	9.0 (1.5)	8.6 (2.2)	< 10^–7^	0.54	0.70
	Fast	4.5 (0.3)**	4.4 (0.4)**			
VLF_p_ (au^2^)	Slow	0.03 (1.23)	0.04 (1.27)	0.35	0.20	0.59
	Fast	0.03 (1.22)	0.06 (1.34)			
LF_p_ (au^2^)	Slow	0.91 (1.02)	0.77 (1.08)	< 10^–7^	0.86	0.21
	Fast	0.04 (1.18)**	0.05 (1.19)**			
HF_p_ (au^2^)	Slow	0.04 (1.19)	0.05 (1.38)	< 10^–7^	0.77	0.35
	Fast	0.90 (1.02)**	0.77 (1.08)**			
Respiratory–cardiovascular coupling						
k_RSP–RRI_ at 25 s	Slow	0.568 (0.245)	0.399 (0.284)	0.017	0.023	0.553
	Fast	0.499 (0.208)	0.336 (0.111)*^,#^			
k_RSP–SBP_ at 25 s	Slow	0.424 (0.099)	0.454 (0.286)	0.58	0.49	0.59
	Fast	0.440 (0.187)	0.391 (0.187)			

Significance by ANOVA and ANOVA on ranks for pNN50 and BRS; *post-hoc* analysis by Fisher's LSD: the * and ** indicate significant differences between times at p < 0.05 and < 0.01; the ^#^ and ^##^ indicate significant differences between groups at p < 0.05 and < 0.01; RRI mean, SBP mean and breathing interval as mean (SD); powers as geometric mean (geometric SD); pNN50 and BRS as median (IQR).

### Respiratory patterns

**Figure 3:**
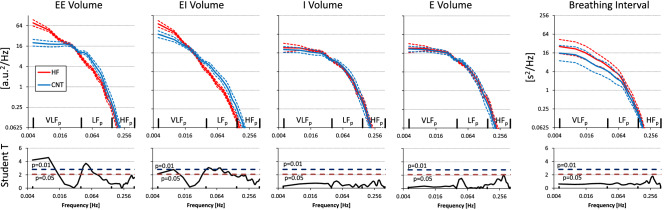
Power spectra of breath-by-breath respiratory series during free breathing. Geometric mean ± geometric sem in HF patients and controls (CNT). The panels below each spectrum show the Student T statistics between groups at each frequency: the horizontal dashed lines are the 0.05 and 0.01 significance thresholds. From left to right: spectra of end-expiratory (EE), end-inspiratory (EI), inspiratory (I), and expiratory (E) volumes, and of breathing intervals.

During free breathing, HF patients showed a respiratory pattern different from controls because of an increased VLF_p_ component (Fig. 2; Table 2). This was due to a larger modulation of both end-inspiratory and end-expiratory volumes, while tidal volumes and breathing intervals were similar to those of controls (Fig. 3).

### Coherence between RRI and SBP

**Figure 4:**
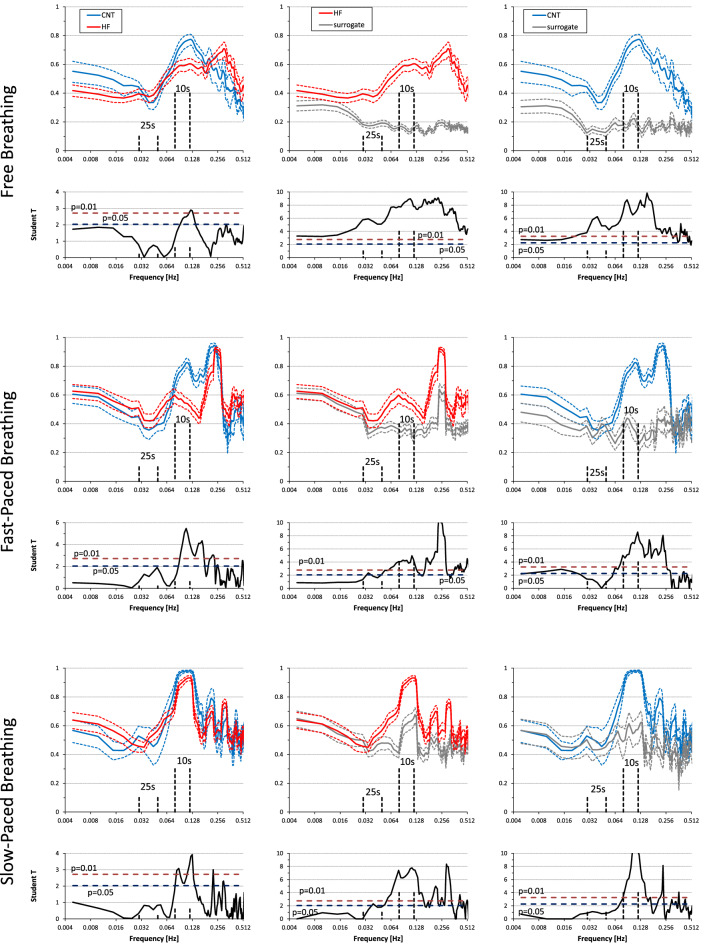
SBP–RRI coherency spectra during free, fast-paced, and slow-paced breathing. Coherency spectra as mean ± sem. Below each spectrum, the Student T between groups is shown at each frequency with horizontal dashed lines indicating the 0.05 and 0.01 significance thresholds. Left panels compare heart failure (HF) patients and controls (CNT): T-values above the dashed lines point out significant differences between groups. Central panels compare original and surrogate coherency spectra in the HF group while right panels compare original and surrogate coherency spectra in the CNT group: T-values above the dashed lines point out coherences significantly greater than 0. Note that the highest coherency occurs at frequencies around the 10-s rhythm and the lowest coherency at frequencies corresponding to fluctuations of about 25 s.

During free breathing, coherence between RRI and SBP was significantly greater than zero at all the frequencies in both controls and HF patients (Fig. 4). In contrast, during fast-paced breathing RRI–SBP coherence was significantly greater than zero only around 0.1 Hz and at the respiratory rate, and during slow-paced breathing only around 0.1 Hz. At 0.1 Hz coherence was lower in HF patients than in controls in all breathing conditions (Fig. 4).

### Coherence between RSP and SBP

**Figure 5:**
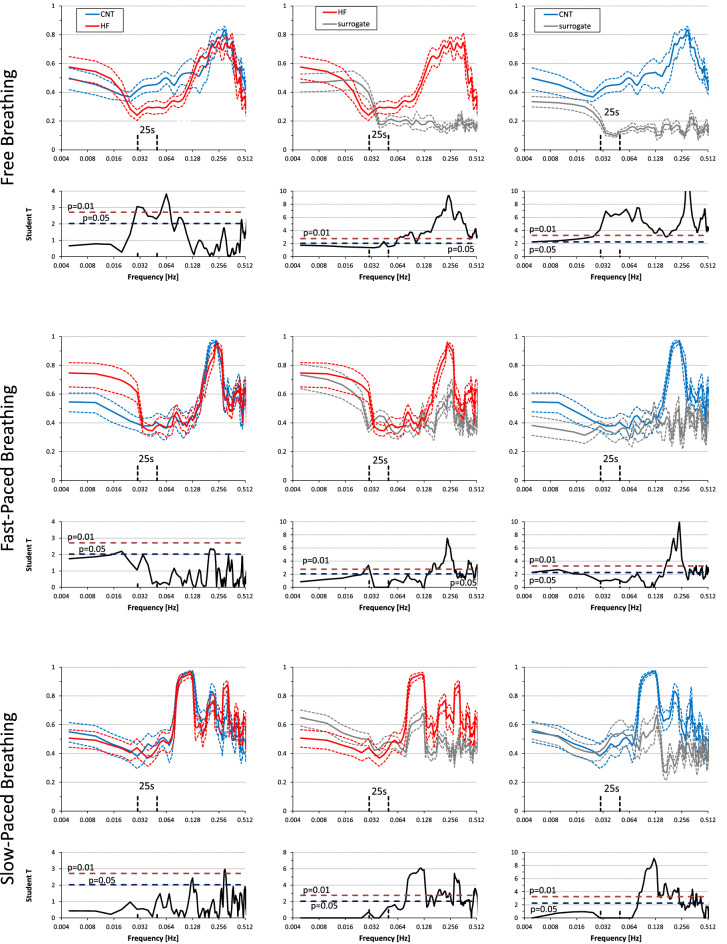
RSP–SBP coherency spectra during free, fast-paced, and slow-paced breathing. Spectra as mean ± sem, with Student T between groups. Left panels compare heart failure (HF) patients and controls (CNT): T-values above the dashed lines point out significant differences between groups. Central panels compare original and surrogate coherency spectra in the HF group while right panels compare original and surrogate coherency spectra in the CNT group: T-values above the dashed lines point out coherences significantly greater than 0. During free breathing, note the significantly greater respiratory-blood pressure coupling around 25 s in the CNT group, and that only in the CNT group, and not in HF patients, the RSP–SBP coherency around 25 s is significantly greater than 0.

During free breathing, coherence between respiration and SBP was significantly greater than zero at all the frequencies in controls while in HF patients the RSP–SBP coherence was greater than zero only at frequencies greater than 0.06 Hz. As a result, RSP–SBP coherency was greater in controls than in HF patients in the very low frequency range (25 s period) (Fig. 5; Table 2). During either fast-paced or slow-paced breathing, RSP–SBP coherence was greater than zero only at the respiratory rate, without differences between groups (Fig. 5).

### Coherence between RSP and RRI

**Figure 6:**
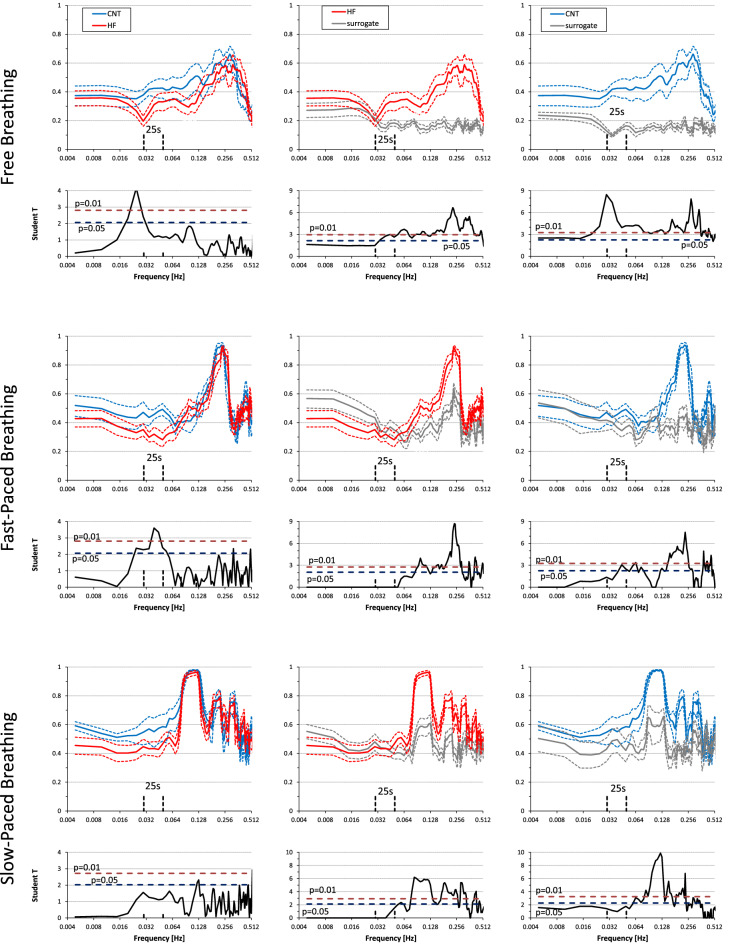
RSP–RRI coherency spectra during free, fast-paced, and slow-paced breathing. Spectra as mean ± sem, with Student T between groups. Left panels compare heart failure (HF) patients and controls (CNT): T-values above the dashed lines point out significant differences between groups. Central panels compare original and surrogate coherency spectra in the HF group while right panels compare original and surrogate coherency spectra in the CNT group: T-values above the dashed lines point out coherences significantly greater than 0.

During free breathing, RSP–RRI coherence was greater than zero at all the frequencies in controls and above 0.03 Hz in HF patients (Fig. 6). This resulted in RSP–RRI coherence greater in controls than in HF patients in the very-low-frequency range but not for oscillations with period around 25 s (Fig. 6; Table 2). During fast-paced breathing, the RSP–RRI coherence around 25 s was greater than zero in controls only and thus it resulted significantly lower in HF patients than in controls (Table 2). During slow-paced breathing the RSP–RRI coherence around 25 s did not differ between controls and patients (Fig. 6; Table 3).

### Baroreflex function

Baroreflex sensitivity was markedly influenced by the pattern of respiration. In HF patients BRS was significantly lower during fast-paced breathing at 12–15 b/min than during free breathing (Table 2), but significantly higher during slow than fast paced breathing (Table 3). In controls, instead, BRS changed only during slow breathing (6 b/min). At this respiratory rate in fact BRS was significantly higher than that observed in HF patients (Table 3). No different BRSs were instead observed during fast paced breathing or free breathing between controls and HF patients (Table 2). The 10-s coherence between SBP and RRI, on the other hand, was significantly lower in HF patients than in controls in all breathing conditions (Table 2), even if slow breathing markedly increased the SBP–RRI coherence at 10-s in both groups (Table 3).

### Spectral analysis: free breathing

During free breathing, VLF_p_ and HF_p_ of both RRI and SBP did not differ significantly between groups while LF_p_ of both RRI and SBP was significantly lower in HF patients than in controls (Fig. 2; Table 2).

### Spectral analysis: free vs. fast-paced breathing

In controls, VLF_p_ and LF_p_ of both RRI and SBP were significantly lower during fast-paced than free breathing while HF_p_ was significantly higher (Table 2). In HF patients, instead, VLF_p_ and LF_p_ of RRI but not of SBP were significantly lower during fast-paced than free breathing and only HF_p_ of SBP but not of RRI was significantly higher during fast-paced breathing (Table 2).

### Spectral analysis: slow vs. fast-paced breathing

The low-frequency power of RRI and SBP was significantly higher during slow-paced than fast-paced breathing in both groups (Table 3); however, LF_p_ during slow breathing was significantly lower in HF patients than in controls (Fig. 2; Table 3). Moreover, VLF_p_ of RRI was significantly higher during slow-paced than fast-paced breathing in controls but not in HF patients (Table 3).

## Discussion

Our study provides three main findings: (1) HF patients showed a pattern of respiration different form that of controls characterized by a greater modulation of ventilation in the VLF range; (2) during free breathing baroreflex sensitivity of HF patients was not different from that of control subjects; (3) the baroreflex of HF patients was sensitive to respiratory pattern and withdrawing respiratory modulation in the VLF range decreased baroreflex sensitivity. These findings will be discussed separately.

### Respiratory pattern

In our HF patients, modulation of respiratory volumes in the very low frequency range was increased. In particular, we observed an increased modulation of end expiratory and end inspiratory volumes at a constant tidal volume. A cyclic stimulation of the chemoreceptors is needed to make respiration change in an oscillatory way, a demonstration being the oscillatory ventilation pattern in the low and very-low-frequency range brought about by spontaneous cyclic changes in the partial pressure of carbon dioxide (PaCO_2_)^22–25^. The increased oscillatory ventilation in the very low frequency range observed in HF patients could be therefore the result of an increased and slower stimulation of the chemoreceptors. Indeed the neuro humoral activation observed in our HF patients^26^ and the increased circulation transit time^27^ described in HF patients could have increased and slowed the stimulation of the chemoreceptors. Whatever the mechanism this pattern of ventilation could have beneficial effects as modulation of respiration in the slow frequency range has been shown to improve alveolar ventilation and respiratory efficiency^28,29^. Moreover, it has been shown that in HF patients a greater modulation of respiratory volumes produces greater swings in intrathoracic pressure that can increase venous return and stroke volume^30^.

### Baroreflex function

During free breathing baroreflex sensitivity of HF patients was not significantly different from that of healthy control subjects. This is unexpected as an impaired baroreflex function has been previously described in presence of an increased sympathetic activity that is a hallmark of HF^26,31,32^. However, our HF patients showed only a mild activation of the sympathetic nervous system and a preserved modulation of heart rate by the parasympathetic nervous system (pNN50 and respiratory sinus arrhythmias) at rest. NT-proBNP and catecholamine plasma concentrations of our patients were in fact below the levels observed in patients with an advanced stage of heart failure^26,33,34^, neurohumoral activation being attenuated by treatment with beta-blockers and inhibitors of the activity of the renin-angiotensin system (Table 1) that have been shown to improve BRS and to reduce sympathetic nerve activity^31,35^. This only mild neurohumoral activation probably not only reduced the negative effects of sympathetic activation on the baroreflex but allowed the baroreflex function to be modulated by the respiratory pattern. In this context a clear finding of our study is that very slow cyclical changes in respiratory volumes were able to improve baroreflex sensitivity of HF patients possibly via an increased vagal modulation of heart rate through activation of the stretch receptors^36^ and a reduced chemoreflex sensitivity and therefore of the negative effect of chemoreceptors on the baroreflex as previously reported^37^. Indeed, during modulation of respiration in the very low and low frequency ranges we observed higher values of very low and low frequency powers of RRI, which are recognized indices of vagal modulation of heart rate^17,38^. The recognition that baroreflex sensitivity is highly sensitive to breathing pattern and that there is a breathing pattern able to improve baroreflex sensitivity is clinically relevant because baroreflex sensitivity is an independent risk factor in HF patients and its increase has been shown to be linked to less life-threatening arrhythmias^39^ and better prognosis^31^. In our study a breathing-dependent modulation of baroreflex-heart rate control was also shown by another index of baroreflex function, i.e., coherence between SBP and R-R interval at 0.1 Hz, which at variance from the previous index, was more clearly impaired in HF patients than in control subjects and it did show an increase during slow breathing. As coherence between SBP and RRI is the result of concomitant modulation of heart rate and peripheral vascular resistances a possible explanation for the decreased coupling between SBP and RRI in HF patients is the reduced capacity of HF patients to modulate peripheral resistances perhaps due to the combined effects of increased sympathetic tone^40^ and reduced vessel distensibility, which is in part also sympathetic dependent^41^. It should be added that in HF patients the increase of 0.1 Hz SBP–RRI coherence during slow breathing was accompanied by an increase of the low frequency power of SBP. This justifies the hypothesis that respiratory related mechanical changes in blood pressure could have entrained the autonomically induced changes leading to an increased amplitude of blood pressure oscillations and a greater engagement of the baroreflex. This would imply that the reduced baroreflex function observed in HF patients derives at least in part from a reduced engagement of the baroreflex because of reduced oscillations in blood pressure.

### Cardiovascular–respiratory coupling

A tight relationship between respiratory and cardiovascular oscillations was present in healthy subjects. Moreover, respiratory oscillations in the very-low and low frequency ranges have been shown to be responsible of a significant part of heart rate and blood pressure oscillations. It is possible therefore that in healthy subjects respiration by modulating the activity of the sympathetic and the parasympathetic nervous systems to the heart and to the blood vessels^36,42–44^ optimizes blood flow distribution in relation to respiratory activity and therefore improves ventilation–perfusion matching^45,46^. In our HF patients, respiration was still able to modulate the RR interval in the very-low and low frequency range, as in healthy subjects, but it lost the capacity to modulate blood pressure in the same frequency range. As breathing in the low frequency range improved cardio-respiratory coupling in HF patients but it did not have any effect on SBP–respiration coupling it could be that the increased modulation of respiration in the very-low frequency range had a positive effect on the cardiorespiratory coupling but not on the coupling between blood pressure and respiration. This, as previously observed, could be due to a reduced ability to modulate the autonomic drive to the blood vessels because of an increased sympathetic tone and because of a less compliant vascular system.

The breathing pattern depends on chemoreceptor activity^22,47,48^ and HF patients show hyperactivation of both central and peripheral chemoreceptors^49^. This is exemplified by HF patients with severe disease in whom strong chemoreceptor activation (due to the disease severity and the marked sympathetic activation) is associated with periodic breathing^50^. Our study provides the first description of the breathing pattern and related influence in HF patients with a less severe disease and a lower neurohumoral activation. We show that HF patients with a less severe disease have a breathing pattern different from periodic breathing (no changes in tidal volume), but also different from a normal pattern. In these patients the pattern of respiration we have studied produces beneficial effects on the autonomic control of heart rate. However, the control that both respiratory activity and the baroreflex exert on blood pressure remains in HF impaired.

### Limitations

We should acknowledge two limitations. First, our observation that fast-paced breathing is associated with a reduction of both the respiratory VLF_p_ and BRS does not definitely prove a causal link between the amplified fluctuations of end-inspiratory/expiratory volumes at the slower frequencies and the improved baroreflex function, as an effect of paced breathing on BRS cannot be excluded. Future studies controlling the respiratory VLF_p_ with methods different from paced breathing might strengthen our conclusions. Second, we estimated the spontaneous baroreflex sensitivity by using one of the more validated methods in the literature, the transfer function technique. However, transfer-function estimates might be influenced also by the feed-forward coupling between RRI and SBP or by cardio-pulmonary reflexes. More sophisticated methods taking into account these aspects^51,52^ may help to better understand the mechanisms underlying our results.

### Conclusions

HF patients with moderate activation of the sympathetic nervous system show an increased modulation of ventilation in the very low frequency range. This pattern of ventilation has positive effects on baroreflex control of heart rate and on cardio respiratory coupling.

## Data Availability

The data supporting the main findings of this study are available in the Zenodo repository at https://doi.org/10.5281/zenodo.6821950 with access granted on justified request to researchers meeting the criteria for access to confidential data due to the hospital research policy and restrictions requested by the ethical committee.
